# Of potential new treatment targets and polythetic approach in meningoencephalitis of unknown origin: a review

**DOI:** 10.3389/fvets.2024.1465689

**Published:** 2024-10-15

**Authors:** Jasmin N. Nessler, Andrea Tipold

**Affiliations:** Department of Small Animal Medicine and Surgery, University of Veterinary Medicine Hannover, Foundation, Hannover, Germany

**Keywords:** canine (dog), meningoencephalitis of unknown origin (MUO), diagnostic, clinical signs, immunology

## Abstract

Meningoencephalitis of unknown origin (MUO) represents an umbrella term for inflammatory, non-infectious central nervous system (CNS) diseases in dogs. Current therapeutic approaches, involving long-term glucocorticosteroid use, often fail to provide adequate relief or cure, and the effectiveness of additional immunosuppressive medications remains uncertain. Future advancements in MUO treatment may benefit from patient-specific therapies, potentially enhancing treatment precision, efficacy, and minimizing side effects. However, significant challenges impede this progress, including ambiguity in MUO subtype classification, uncertainties regarding the autoimmune nature vs. infectious triggers, and the lack of reliable diagnostic biomarkers. Clinical heterogeneity and overlapping signs with other encephalopathies further complicate diagnosis and treatment. This review gives an overview about diagnostic findings and immunological features of MUO. It advocates for a more overall characterization of MUO by using a polythetic system to better characterize MUO subtypes, identify immunological treatment targets, and establish a conceptual foundation for future therapeutic trials. Addressing these themes may lead to more effective and less burdensome treatments, improving the quality of life for dogs afflicted with MUO and their owners.

## 1 Introduction

Meningoencephalitis of unknown origin (MUO) is an inflammatory, non-infectious disease of the central nervous system (CNS) in dogs (1, 2). MUO primarily functions as an umbrella term, frequently utilized for diagnostic purposes in the absence of histopathological confirmation and specific classification (3, 4). Most commonly mentioned subtypes encompass granulomatous meningoencephalomyelitis (GME), necrotizing meningoencephalitis (NME), and necrotizing leukoencephalitis (NLE) (4) often summarized as necrotizing encephalitis (NE) (2). Less common entities, such as eosinophilic meningoencephalitis, greyhound encephalitis, idiopathic cerebellitis, and autoantibody encephalitis are occasionally excluded from the MUO category in certain literature (4). Steroid-responsive meningitis-arteritis (SRMA), a predominant neutrophilic meningitis and vasculitis, remains a distinct entity and is not subsumed within the MUO terminology (4, 5).

MUO lacks an identifiable infectious trigger and typically respond positively to immunosuppressive therapy, primarily through long-term glucocorticosteroid application (4, 6–9). Despite a variable and sometimes insufficient response to therapy and the high incidence of side effects, the treatment regimen has remained largely unchanged for the last 10 to 20 years (9).

The current state of therapeutic approaches for MUO in cats and dogs is unsatisfactory. Long-term therapy with glucocorticosteroids improves the outcome in treated patients compared to untreated patients; but the efficacy of commonly used additional immunosuppressive medication remains inconclusive (9–11). This treatment results in often intolerable burden of side effects and failing to sufficiently provide long-term relief from clinical manifestations, let alone cure the disease.

For the future, a patient-specific therapy would be desirable as it might have several advantages (12):

Precision medicine: Patient-specific therapy might allow for a more precise and targeted approach to treatment. By understanding the unique characteristics of the pet's autoimmune CNS disease, medications and interventions could be customized to address the specific mechanisms driving the condition. Tailoring treatment to the individual patient's needs could enhance the effectiveness of interventions while minimizing potential side effects.

Optimized efficacy: Different dogs may respond differently to standard treatments. Personalized therapy takes into account the specific molecular and cellular factors contributing to the autoimmune response and the stage of the disease, increasing the likelihood of a more effective treatment outcome.

Minimized side effects: Standard treatments for autoimmune diseases often involve pan immunosuppression that can affect various bodily functions (13). Tailoring therapy to the individual patient might help minimize unnecessary exposure to medications, reducing the risk of adverse effects.

Improved quality of life (QoL): By customizing treatment to the unique characteristics of the dog's autoimmune CNS disease, veterinarians could optimize the balance between suppressing clinical signs and preserving the dog's overall wellbeing. This can lead to an improved quality of life for the patients and their owners.

Development of satisfactory and patient-specific treatment necessitates a profound understanding of the etiopathogenesis of MUO, identifying potential treatment targets. Three significant challenges hinder this understanding:

First, the ambiguity persists regarding whether all MUO subtypes constitute a single disease entity or if histopathological subtypes represent distinct independent diseases with varying etiopathogenesis (2).

Second, uncertainties remain regarding the clear autoimmune nature of these diseases or the potential involvement of a relevant infection triggering and initiating excessive inflammation (9).

Third, there is a lack of consensus on how to diagnose MUO or its subtypes ante mortem without histopathological confirmation via biopsy, which is an expensive, time-consuming, and potentially risky procedure, not routinely employed. Currently, no single biomarker or combination thereof reliably establishes the diagnosis of MUO or distinguishes between subtypes (14). Clinical signs and laboratory findings of dogs with MUO exhibit heterogeneity and occasionally, MRI and/or cerebrospinal fluid (CSF) findings are normal: Abnormal MRI with normal CSF is present in 9%, normal MRI and abnormal CSF in 6%, and normal MRI and CSF in 3% of dogs with MUO (15), in general 13% of dogs with MUO show normal CSF (4). Dogs of any breed and age may be affected, challenging breed specific predisposition patterns. Clinical signs and findings also overlap significantly with other encephalopathies (16). Strict adherence to previously suggested definitions of MUO would exclude a considerable number of affected dogs and could even include dogs with other diseases (4).

To advance new and more effective therapies with less side effects for patients with MUO, this review aims to lay the conceptual foundation for future therapeutic trials by addressing the following overarching themes:

- Characterizing diverse MUO subtypes beyond the conventional subtypes of GME and NE observed in dogs.
- Identifying potential treatment targets within the immunological disease process.
- Advocate for a more overall characterization of MUO by using a polythetic approach.

## 2 History of MUO

First described in 1968, GME was initially referred to as reticulosis and predominantly considered neoplastic (17). However, in the subsequent decades, particularly in the 70s and 80s, it became increasingly evident that this entity is more appropriately characterized as an inflammatory disease (18–23). Despite extensive investigations, no infectious agents were identified, and the underlying cause remained unclear (18, 19). Various theories were postulated, including the suggestion that GME might represent an aberrant reaction to canine distemper virus infection or vaccination, a retrovirus infection stemming from vaccine contamination, or involvement of other viral, parasitic, or infectious agents (24). None of these theories could be substantiated through immunohistochemical examinations, polymerase chain reaction (PCR) analyses, viral inoculation, or microbiological cultures, failing to reveal a conclusive infectious agent triggering the inflammatory response (8, 24–26). Early reports also proposed an overstimulation of the immune system due to the antiparasitic drug levamisole (27) or a T-cell-mediated delayed-type hypersensitivity reaction as potential causes for GME (24, 28); however, none of these hypotheses could be definitively proven.

Subsequently, NME- in Pug dogs often referred to as Pug dog encephalitis due to its strong breed disposition (29)- and NLE in Yorkshire Terriers were documented (6). Once again, no infectious agents were identified (25, 30).

Clinical examination, CSF analyses, and computed tomography (CT) scans proved insufficient in differentiating between the various histopathological subgroups (4, 31). As a result, these meningoencephalitides were amalgamated for clinical purposes and described under diverse names such as idiopathic inflammatory brain disease, non-infectious meningoencephalitis, and sterile meningoencephalitis, among others (1, 2, 4, 32). Over time, MUO has emerged as the prevailing and widely accepted term (4, 33). Presently, MUO stands as one of the most common encephalitis, accounting for 52%−69% of dogs with encephalitis (5, 34).

Despite the array of names and classifications, therapeutic approaches have shown limited evolution in recent years (4, 9). Early manuscripts outlined the utilization of glucocorticosteroid therapy in individual dogs diagnosed with MUO (19). Given the variability in outcomes, additional immunosuppressive drugs such as mycophenolate mofetil, leflunomide, cyclosporine, azathioprine, and even radiation protocols were introduced alongside glucocorticoids (35–44). While the available data may exhibit some contradictions, on the whole, none of these therapies seems to demonstrate superiority over others in terms of efficacy and side effect profiles (9). Furthermore, uncertainties persist regarding whether combination therapy offers a significant advantage over the use of glucocorticoids alone (11, 45, 46).

To the author's knowledge, there are no studies evaluating the effectiveness or side effect profiles of other immunosuppressive drugs without concurrent application of glucocorticosteroids in MUO (9). Additionally, there is only one comparative study assessing combined therapy with glucocorticosteroids and an additional immunosuppressive drug against treatment with glucocorticosteroids alone in a prospective, double-blinded approach (9, 11).

Presently, one of the most commonly employed treatment regimens entails the administration of parenteral cytarabine in conjunction with long-term glucocorticosteroids (10). However, the prolonged use of glucocorticosteroids is associated with numerous side effects, including polyuria/polydipsia, gastrointestinal symptoms, alopecia, calcinosis cutis, and others (13, 47). These side effects significantly impact QoL of the dog and the owner (48). Attempts to reduce glucocorticoid doses often result in deterioration or relapse of clinical signs (40). Even with appropriate doses, treatment frequently yields insufficient clinical improvement, and the mortality rate of MUO can reach up to 30%−56% within 100 days after diagnosis (33, 40, 49).

For a detailed review on therapeutic options used at the moment and treatment results, we would like to refer the reader to the latest reviews, for example by Jeffery and Granger (9).

## 3 Clinical diagnosis of MUO

Clinical signs vary with lesion localization and reflect focal or multifocal lesions in the CNS, commonly involving seizures, ataxia, proprioceptive deficits, vestibular signs, blindness, and multiple cranial nerve deficits, sometimes accompanied by head or neck pain (24). Systemic signs like fever are rare (4). GME mainly causes signs of lesions in the forebrain, brainstem, or both (4, 19) and blindness in the ocular variant (50, 51). Large breed dogs presented significantly more often with decreased mentation compared to small breed dogs (33). NE mainly causes signs of forebrain lesions with seizures (6, 29, 30, 52, 53). Rarely, GME can occur as meningomyelitis and cause paresis and spinal ataxia, with approximately 9.5%−13% involving the spinal cord only (4, 54, 55).

To assess the severity of clinical signs objectively, two neurodisability scales (NDS) have been developed so far (37, 56) which attribute scores to different clinical signs. While Goncalves et al. (5) showed good interobserver agreement in prospective cases, this was worse in retrospective evaluation of patient records. Therefore the authors do not encourage the use of this score for retrospective data (56). Which of the scores is superior at any level was not evaluated so far, but it seems they show good correlation (57).

Pug dogs with NME tend to be younger (median 18 months) than other dogs with NE (2–4 years), and dogs with GME tend to be older (median 55 months; range: 6 to 144 months) when the first signs of encephalopathy occur (2, 4, 53).

Diagnostics may involve advanced imaging, preferably magnetic resonance imaging (MRI), of the brain and/or spinal cord, CSF analysis, and exclusion of regional infectious agents (4).

MRI findings can vary significantly, but the classic presentation typically consists of multifocal, intra-axial, ill-defined lesions with mild mass effect and inhomogeneous contrast enhancement (33). But MRI findings in MUO can be normal in up to 7%−9% (4, 15).

In GME, MRI reveals focal, multifocal, or diffuse T2-weighted (T2w) and fluid attenuating inversion recovery (FLAIR) hyperintense lesions in the forebrain, brainstem, or cerebellum in both white and gray matter. The degree of contrast enhancement in the CNS parenchyma varies, with little to minimal contrast enhancement of the meninges (58).

MRI findings for NE differ slightly among affected breeds but exhibit significant overlap. In Yorkshire Terriers with NE, lesions are mostly multifocal, uni- or bilaterally asymmetrical lesions in the forebrain, including the diencephalon. Predominantly, these lesions occur in the periventricular and subcortical white matter, often sparing the cortical gray matter (59, 60). The brainstem is often less severely affected, while the cerebellum and spinal cord typically remain unaffected. These lesions are usually T2w and FLAIR hyperintense, although in more chronic cases, FLAIR signal might be suppressed (59, 60). Contrast enhancement is mostly mild and inhomogeneous to patchy (59).

MRI in NE in Pug dogs and Chihuahuas exhibit multifocal or diffuse, asymmetrical forebrain lesions, most severe in occipital and parietal lobes, with the frontal lobes less frequently affected. Diencephalic lesions are less common, and brainstem or cerebellar lesions are possible but rare (61). The border between white and gray matter is often blurred, and most lesions are present in the gray matter. Lesions are usually T2w and FLAIR hyperintense, although in more chronic lesions, FLAIR signal might be suppressed (59, 60). Contrast enhancement is mostly mild and inhomogeneous to patchy (59).

CSF shows increased protein and predominantly mononuclear pleocytosis, but mixed to neutrophilic pleocytosis, albuminocytologic dissociation are not uncommon, and up to 22% of dogs with MUO display normal CSF (4). C-reactive protein (CRP) in CSF is not significantly different from healthy dogs (62).

Blood tests and extracranial findings are generally within physiological limits, which is often in contrast with infectious meningoencephalitis. For instance, in cases of neosporosis, blood creatine kinase activity may be elevated (63).

Based on clinical signs, blood tests, MRI, and cerebrospinal fluid findings, as well as the patient's place of residence and travel history, possible infectious agents should be excluded with appropriate investigations. There can be no uniform recommendation for all cases, as the appropriate pathogen investigations can vary significantly depending on the individual case.

For research purposes, currently, the most widely adopted inclusion criteria to diagnose MUO encompass the following points (4):

- Age: > 6 months.
- Evidence of a multifocal CNS disease demonstrated by either multifocal or diffuse lesions suspected after the neurological examination and multiple, single, or diffuse intra-axial hyperintense lesions on T2w MR images, or a unifocal lesion suspected after the neurological examination and multiple or diffuse intra-axial hyperintense lesions on T2w MR images.
- CSF analysis should be hypercellular, with >50% mononuclear cells (preferably monocytes/lymphocytes).
- Infectious diseases should be ruled out.

The accuracy of these inclusion criteria has not been thoroughly examined and there is debate among researchers on these points. Recent reports about MUO in dogs younger than 6 months might additionally question the age at inclusion (64).

Diagnosis can be complicated by overlapping clinical, MRI, and CSF findings with other conditions such as neoplasia (16). Confirming the diagnosis requires histopathological confirmation of sterile inflammation (65). Therefore, the identification of the histopathological subtype of GME or NE through clinical diagnosis alone is limited. Due to this limitation, a uniform definitive consensus on the diagnosis of MUO for clinical or research purposes has not been established (14).

## 4 Prognostic factors of MUO

Prognosis is guarded with MUO, and various diagnostic indicators appear to be linked to mortality risks (2, 3, 33, 35, 40, 46, 55, 66, 67). Clinical manifestations such as reduced mentation, seizures, and signs related to multifocal or caudal cranial fossa lesions are indicative of a less favorable prognosis (55). In general, severity of clinical signs expressed as higher clinical NDS seem to be associated with 1-week-survival but not with long term survival in dogs with NE (56, 57).

Additionally, dogs with higher body weight or advanced age and juvenile dogs tend to face a worse outcome (33, 64, 67).

Elevated and/or neutrophilic CSF cell counts, and hyperlactatemia are correlated with shorter survival times (66, 67).

MRI lesions seem to be correlated with prognosis to some degree. Severe MRI findings, such as the loss of the CSF signal of the cerebral sulci and foramen magnum herniation, are associated with an increased risk of mortality (40). High T2w lesion burden might be correlated with worse long term prognosis (68) while unremarkable MRI findings seem to be associated with better prognosis (69). Increased contrast enhancement might be correlated with increased risk of relapse (68).

## 5 MUO in cats

MUO is not limited to dogs, with infrequent reports of its occurrence also in cats (1, 70). Additionally, although not explicitly named “MUO,” feline meningoencephalitides have been documented from authors proposing an infectious agent without having been able to identify it (71–73). A similar scenario is observed in Staggering disease: The term Staggering disease describes a clinical syndrome caused by non-suppurative, lymphohistiocytic meningoencephalomyelitis (74, 75). Initially believed to result from a viral infection, Borna virus (BoDV-1) was evaluated as a potential causative agent (76). While experimental infections demonstrated BoDV-1-induced neurologic disease in domestic cats (77), consistent detection with independent diagnostic methods proved elusive (78, 79). Consequently, BoDV-1 is no longer considered the causative agent for staggering disease, leaving it classified as a meningoencephalitis with an unknown trigger for some time. Recent evidence, however, suggests that some cases of staggering disease may be caused by Rustrela virus (RusV), as it has been detected in several affected cats (80). Nonetheless, there are still cats with clinical signs of MUO or staggering disease where no infectious agent, including RusV, can be identified (80, 81). This suggests that staggering disease may represent a spectrum, with both unknown (MUO) and infectious causes, depending on the individual case.

In cats, reports on histopathologically confirmed MUO in combination with clinical signs are rare (81). The median age of cats is 7 years, older than the median age of cats typically presented to the clinic for infectious encephalitis (81). The breed distribution of cats with MUO is comparable to the general clinical population, while pedigree cats appeared to be more common among cats with infectious encephalitis (81). Besides acute or chronic neurological signs, systemic signs of illness or blood leukocytosis were frequently present. CSF changes appeared subtle, with albuminocytologic dissociation being the most common finding. Histopathology revealed a multifocal, lympho-histiocytic inflammation in the CNS (81).

Diagnostic and therapeutic strategies for MUO in cats primarily arise from canine MUO research; treatment commonly involves the use of prednisolone (70). However, therapeutic guidance remains largely anecdotal.

## 6 The search for infectious agents

In canine MUO, several attempts failed to reveal any underlying infectious diseases (8, 24–26, 82). Although some potential infectious agents like bacteria or viruses were identified in individual animals, none were consistently detected across the entire patient cohort, ruling out that they are the underlying cause of MUO (8, 24, 26, 82). This strengthens the hypothesis that MUO might not be triggered by a specific infectious agent but is a genetic disease. On the other hand, RusV was detected in the CNS of cats initially diagnosed with MUO (80). RusV ribonucleic acid (RNA) and antigen were shown by metagenomic sequencing, real-time quantitative polymerase chain reaction (PCR), *in-situ* hybridization, and immunohistochemistry in brain tissues of 27 out of 29 cats with non-suppurative meningoencephalomyelitis without a previously identifiable cause (80). Screening of possible reservoir hosts in Sweden revealed RusV infection in Wood mice (*Apodemus sylvaticus*) (80). RusV is a relative of the rubella virus and associated with encephalitis in various mammalian hosts, including Wood mice, lions, and wallabies (83–85). It demonstrates a broad host spectrum and extensive geographic distribution, raising the possibility of its involvement in neuropathologies across diverse mammalian species, potentially even humans (80, 83–86).

However, the absence of RusV in some cats with lymphohistiocytic meningoencephalitis shows that MUO might still be a distinct entity in felines, albeit seemingly less prevalent than in canines (80). The divergence in results between cats and dogs with MUO suggests difference in the underlying pathogenic or genetic mechanisms between the two species. While in MUO in dogs a lack of identifiable infectious agents and breed predispositions (4, 82) point toward autoimmune etiology, the prevalence of RusV in cats implies a more prominent role of infectious agents in feline CNS inflammation.

The interspecies difference is most probably influenced by various factors. Lifestyle distinctions, such as dogs being more leash-restricted and less prone to consuming prey, may reduce their exposure to infectious agents (87). In contrast, the outdoor habits of cats, including hunting, may increase their vulnerability to pathogens like RusV.

The broader exposure to antigens during their outdoor pursuits could contribute to the development of a more diverse and potentially regulated immune system in cats, potentially reducing the susceptibility to autoimmune disorders: The so called “hygiene hypothesis” proposes that reduced early-life exposure to infections and a cleaner, more sanitized environment may contribute to the increased prevalence of autoimmune diseases (88). This theory suggests that limited microbial exposure early in life may lead to an improperly regulated immune system, increasing the risk of allergic and autoimmune diseases (88, 89). Additionally, gastrointestinal parasite infections are immune modulatory (90), and the potential predisposition of cats to such infections due to their outdoor lifestyle might save them from overreacting immune responses, although, no discernible difference in deworming practices between cats and dogs emerges from existing studies (87, 91).

In human medicine, there is a latitude difference in prevalence of Multiple sclerosis (MS): Individuals residing in countries closer to the equator during their initial years of life exhibit a lower incidence of MS possibly related to sunlight exposure and higher vitamin D levels (92, 93). Pets like dogs have a different mechanism of synthesizing vitamin D (94, 95), but a certain influence from exposure to sunlight might still be possible. The outdoor lifestyle of cats may influence the developing immune system differently compared to the immune system of young dogs mostly held indoors. It might be possible that early exposure to sunlight is beneficial to developing a healthy immune system (89).

Most likely, the cause for an increased prevalence of autoimmune CNS disease in dogs compared to cats is the difference in the genetic background (6, 29, 96–98). Cats, especially the more common European shorthaired cats, may be less inbred than dogs, leading to a reduced genetic predisposition to MUO.

## 7 Genetic base of MUO

A genetic basis for MUO is highly probable, and distinct breed-specific patterns are evident (6, 52, 55, 59). NE predominantly affects toy breeds, while NME is prevalent in breeds such as Pug dogs, Maltese, or Chihuahuas (29, 52, 99). On the other hand, NLE is more commonly observed in Yorkshire Terriers and French Bulldogs (6, 30, 59). The distribution of GME appears more heterogeneous, primarily affecting toy and terrier breeds, but approximately one third of affected dogs belong to larger breeds with a body weight exceeding 15 to 20 kg (4, 33, 55, 100).

In the context of genetic predisposition, heritability of NME specific to the Pug dog is 0.67 (101). NME is particularly associated with the Major Histocompatibility Complex II (MHC II) haplotype featuring DRB1-010011, DQA1-00201, and DQB1-01501 (102, 103). Also, Maltese dogs and Chihuahuas seem to be at increased risk to develop MUO with a certain MHC II haplotype (99, 104). MHC II plays a crucial role in antigen presentation and has been correlated with various autoimmune diseases in both canines and humans, including Vizsla polymyositis and MS (105, 106).

## 8 Histopathological findings in MUO and classification of MUO subtypes

MUO mostly serves as an umbrella term, predominantly employed for diagnostic purposes in the absence of histopathological confirmation and classification. Noteworthy subtypes include GME and necrotizing encephalitis (NE) (comprising NME and NLE). Less frequent entities such as eosinophilic meningoencephalitis, greyhound encephalitis, optic neuritis, idiopathic cerebellitis, and other unclassified sterile meningoencephalitides are occasionally excluded from the MUO category in some publications (4).

Histopathological characteristics exhibit specificity for each MUO subtype. GME is classified by asymmetric angiocentric or nodular granulomatous lesions arising from the focal eccentric nodular proliferation of macrophages within histiocytic perivascular cuffs in the Virchow-Robin space, primarily evident in the cerebellum, medulla oblongata, and spinal cord (19–21, 107). NE manifests as non-suppurative perivascular inflammation and necrotic lesions predominantly in the white matter of the cerebrum and brain stem, or the gray matter and meninges of the telencephalon in NLE or NME respectively (6, 29, 30, 107).

GME, NME, and NLE all show a predominance of CD3-positive T cells, along with macrophages and plasma cells. The differences between these MUO subtypes are relatively subtle (108). In NME and NLE, macrophages are frequently observed in the malacic neuroparenchyma, where they likely help remove cellular debris (108). In contrast, in GME, macrophages are more commonly found in the perivascular cuffs, suggesting their role in forming granulomatous lesions as part of the immune response (108).

In NME and NLE, CD3-positive T cells adhere to astrocytes in malacic regions, with this interaction occurring in different areas: in the cortex for NME, and in the white matter for NLE and GME (108). Furthermore, astrocytes stain positive for IgG in NME and NLE (but not in GME), in distinct regions—protoplasmic astrocytes in the cortex in NME and fibrous astrocytes in the white matter in NLE (108, 109). This suggests that different target structures may be involved in the inflammatory processes of NME and NLE.

However, the distinctiveness of histopathological features is not universally observed, with frequent overlap between NME and NLE, leading to their collective designation as NE (2).

Although there is general scientific agreement that typical and distinct features regarding age of onset, clinical signs, and histopathologic findings in GME and NE exist, it could be shown that there is more overlap between MUO subtypes and more distinct subtypes than previously known.

In one study, we have shown that it is possible to detect concomitant histopathological features of GME and NE in the brain of a single dog: Microscopically, in four dogs, areas of marked necrosis were evident in the cerebral hemispheres, cerebellar white matter, or brain stem with mainly lymphocytic perivascular infiltrates (110). At the same time, all four dogs also had focal or multifocal high-grade angiocentric granulomatous inflammatory lesions in the cerebrum, and rhombencephalon. Meningitis was found in all dogs. Infectious agents were excluded. This study suggests that there might be additionally significant overlaps between GME and NE. Those dogs were dogs from breeds traditionally considered to suffer from NE variants (6, 30, 52, 99, 110).

Additionally, another breed predisposition in Australian Shepherds was described, which experience MUO at a senior age and likely suffer from GME (100). This underscores an age-dependent susceptibility to MUO in Australian Shepherds.

Furthermore, we have discovered an as-yet-undescribed variant of lympho-histiocytic meningoencephalitis with CNS vasculitis of unknown origin (111). Dogs exhibited clinical signs of severe forebrain disease, rapidly progressing to involve the brainstem, ultimately leading to death. Extracranial clinical signs were only mild (111). MRI examination revealed generalized swelling of cerebral gray matter and subsequent features of increased intracranial pressure, as well as signs of cerebellar and brainstem hemorrhage or transtentorial herniation (111). CSF analysis indicated hemorrhage and lymphocytic dominance in cell differentiation. In necropsy, the brains displayed varying degrees of edema, cerebellar herniation, and hemorrhages. Microscopically, the primary findings comprised lympho-histiocytic inflammation in the brain and/or spinal cord with associated vasculitis (111). An infectious causative agent could not be determined. This highlights that MUO exhibits a much more extensive diversity than previously reported.

This raises the question, what factors contribute to the expression of different inflammatory patterns. The current consensus suggests a multifactorial pathogenesis for MUO (9). Some authors propose a genetic predisposition and a triggering factor like an infectious agent or that exogenous antigens activate T-cells cross-reacting with self-antigens, called molecular mimicry (1, 8, 112). However, as no exogenous triggers have been found in the last 60 years in canine MUO (1, 8, 25, 82, 113), it seems more and more likely that there might be none.

Another theory postulates a multistep pathogenesis of autoimmune disease (114). An autoimmune disease might be caused by a failure of immunological self-tolerance caused by multiple inherited and somatic mutations within the immune system (114). According to current knowledge, autoimmune diseases arise when T and B-cells responding to self-antigens cause misguided and over-reactive inflammation (115, 116).

Physiologically, immunological self-tolerance involves multiple control systems to prevent the accumulation of autoimmune lymphocytes. The first step is central immune tolerance, involving the purging of autoimmune cells in the thymus (117, 118). Here, up to 40% of autoreactive cells escape central immune tolerance (117).

Several subsequent mechanisms are involved in the peripheral immune tolerance to limit auto-reactive immune cell responses (114, 116). Peripheral immune tolerance is enforced through cell-intrinsic (inhibitory pathways) and cell-extrinsic (regulatory T-cells = Tregs) mechanisms (118). Tregs, characterized by their anti-inflammatory properties, suppress autoimmune reactions through various means, including the secretion of anti-inflammatory cytokines (IL-10, TGF-β, and IL-35) and induction of apoptosis in effector cells (119, 120).

Intrinsic regulatory mechanisms involve rendering T-cells non-responsive to antigens (anergy), if they engage a MHC molecule on an antigen-presenting cell without concurrent engagement of co-stimulatory molecules (121, 122). Co-stimulatory molecules, upregulated by pro-inflammatory cytokines during acute inflammation, are essential for T-cell activation. An absence of pro-inflammatory cytokines results in the non-expression of co-stimulatory molecules, leading to anergy (122–125). Therefore, auto-reactive T-cells stay inactive although they have contact with “their” auto-antigen as long as no pro-inflammatory reaction is present.

Moreover, anatomical barriers, such as the blood-brain barrier surrounding CNS parenchyma, can impede the interaction between auto-reactive lymphocytes and antigens (126, 127).

The development of autoimmune diseases necessitates the bypassing of several of these regulatory mechanisms. While a singular gene defect has not yet been identified as the causative factor for MUO, the prevailing hypothesis leans toward multigenetic defects (96, 98, 99, 104, 114). This suggests that the failure of multiple safeguard mechanisms contributes to the development of autoimmune diseases. In the context of genetic predisposition, Pugs exhibit a recognized susceptibility, particularly associated with a specific MHC II haplotype (102). Before even presenting any clinical signs of MUO, asymptomatic pugs already display variations in their immune system and in their serum anti-glial fibrillary acidic protein (GFAP) antibodies can be detected (128, 129). GFAP is mainly part of intermediate filaments in the cytoplasm of astrocytes (130). The presence of anti-GFAP antibodies in the periphery means auto-reactive B cells were activated to produce immunoglobulins (131). These findings indicate that antigen presenting cells had contact with GFAP (131). GFAP is mostly expressed intracellularly but can be released into the blood after astrocyte damage where peripheral auto-reactive immune cells might have contact to GFAP and initiate anti-GFAP antibody production (132). On the other hand, peripheral auto-reactive immune cells could have crossed a pre-damaged blood brain barrier (126, 127).

Additionally, asymptomatic Pug dogs with the high-risk MHC II haplotype show low numbers of pro-inflammatory CD4+ cells in peripheral blood as well as high plasma levels of the anti-inflammatory chemokine IL-10 (128). This might be a compensatory mechanism of the peripheral immune tolerance to keep controlled auto-reactive immune cells. Failing mechanisms may lead to clinically apparent NME.

A genetic predisposition to autoimmune diseases may suggest early onset, although this assertion is only partially accurate. SRMA (a suspected immune-mediated meningitis) typically occurs between 3–18 months of age, while Pugs exhibiting NE typically manifest initial signs of central nervous system dysfunction around 18 months of age on average (53, 97). Other breeds susceptible to NE generally present signs at a slightly older age, ranging from 2 to 4 years (4). Dogs affected by GME tend to be even older, with an age range of 4–8 years, and Australian Shepherds are even diagnosed in their senior years (4, 100).

This can be explained by the theory of multistep pathogenesis of autoimmune disease, which includes an explanation of delayed stochastic penetrance, where physiologic mutations in T-cells might lead to auto-reactive cells (114): physiological and unphysiological activation of lymphocytes by antigens triggers clonal lymphocyte proliferation. In this process, T-cell receptors can change from one T-cell generation to the next. Physiological mechanisms of somatic recombination, gene conversion, and somatic mutation constantly equip the T and B-lymphocytes system with different receptors for detecting antigens (114). Those processes generate random rearrangements of gene segments and result in novel amino acid sequences in the antigen-binding regions of immunoglobulins and T-cell receptors to be equipped for novel antigens (114). This increases the possibility that the immune system can detect a wide variety of external antigens. However, more than half of all antigen receptors generated randomly through somatic recombination also possess the capability to recognize self-antigens (114). Consequently, increased lymphocyte activation increases the risk of auto-reactive memory cells that accumulate with advancing age. This partly amplifies the susceptibility to developing autoimmune diseases with increased age.

Due to the overlap of signalment, clinical signs, and histopathological findings, it seems reasonable to talk of MUO as a disease spectrum rather than an umbrella term summarizing different diseases. In addition, it seems reasonable to include all suspected autoimmune CNS diseases and not only GME and NE as some authors do at the moment (4). Classifying the MUO spectrum should also include breed and age of onset rather than histopathological confirmation alone.

## 9 Immunological features of MUO

The immunological properties of MUO remain poorly understood. Frequently, studies tend to group various histological subtypes under either MUO or the broader category of inflammatory brain disease (133–135), complicating the assessment of distinctions between MUO subtypes.

When considering lymphocyte population, GME exhibits a mixed pattern involving both B and T-cells, whereas NE is predominantly T-cell-driven (107). CD3+ cells in the CNS of NE-affected dogs produce interferon (IFN)-gamma, contributing to neuronal necrosis in NE (107). Here, a recent study suggested a mild potential superiority of cyclosporine add-on over other add-on therapies when it comes to prognosis in dogs with NE (60). This could be attributed to cyclosporine reducing IFN gamma (136).

In the CNS parenchyma of dogs with GME, a T-helper (Th) 2-dominated immune response is observed (107). Cluster of differentiation (CD)3+ cells in the CNS of GME-affected dogs produce interleukin (IL)-21, IL-17, and IL-4 (107). Monocytes or microglia in GME release IL-17 within the CNS parenchyma (28, 107). IL-21, a type I cytokine produced by T-cells and natural killer T-cells, inhibits the maturation and function of bone marrow-derived dendritic cells (137). However, IL-21 might act as a “double-edged sword” with both stimulatory and suppressive potential, depending on the context (137).

Immunohistochemically activated astrocytes are visible in all subtypes of MUO (138). In close proximity, anti-Glial fibrillary acidic protein (GFAP) antibodies can be found, which are also present in CSF and serum (129, 139, 140). Although anti-GFAP antibodies can also be found in low levels in dogs with brain neoplasia and other encephalopathies, there is a high possibility that they are involved partly in the pathogenesis of MUO (141). This might give a hint to the occurrence of activated auto-reactive B cells and could potentially trigger autoimmune diseases. Notably, anti-GFAP antibodies can also be detected in asymptomatic pugs (129).

Asymptomatic pugs at risk of developing NME due to a specific MHC II haplotype already show variation in their immune system before clinical signs are evident: They exhibit low numbers of CD4+ cells in peripheral blood (128). CD4+ T-lymphocytes play a pivotal role in antigen recognition (142). Furthermore, they communicate with B-lymphocytes, guiding the production of antibodies (143). Moreover, high plasma levels of the anti-inflammatory chemokine IL-10 are present in asymptomatic pugs with the NME risk MHC II haplotype (128).

### 9.1 Interleukin-31 in MUO

**Figure 1:**
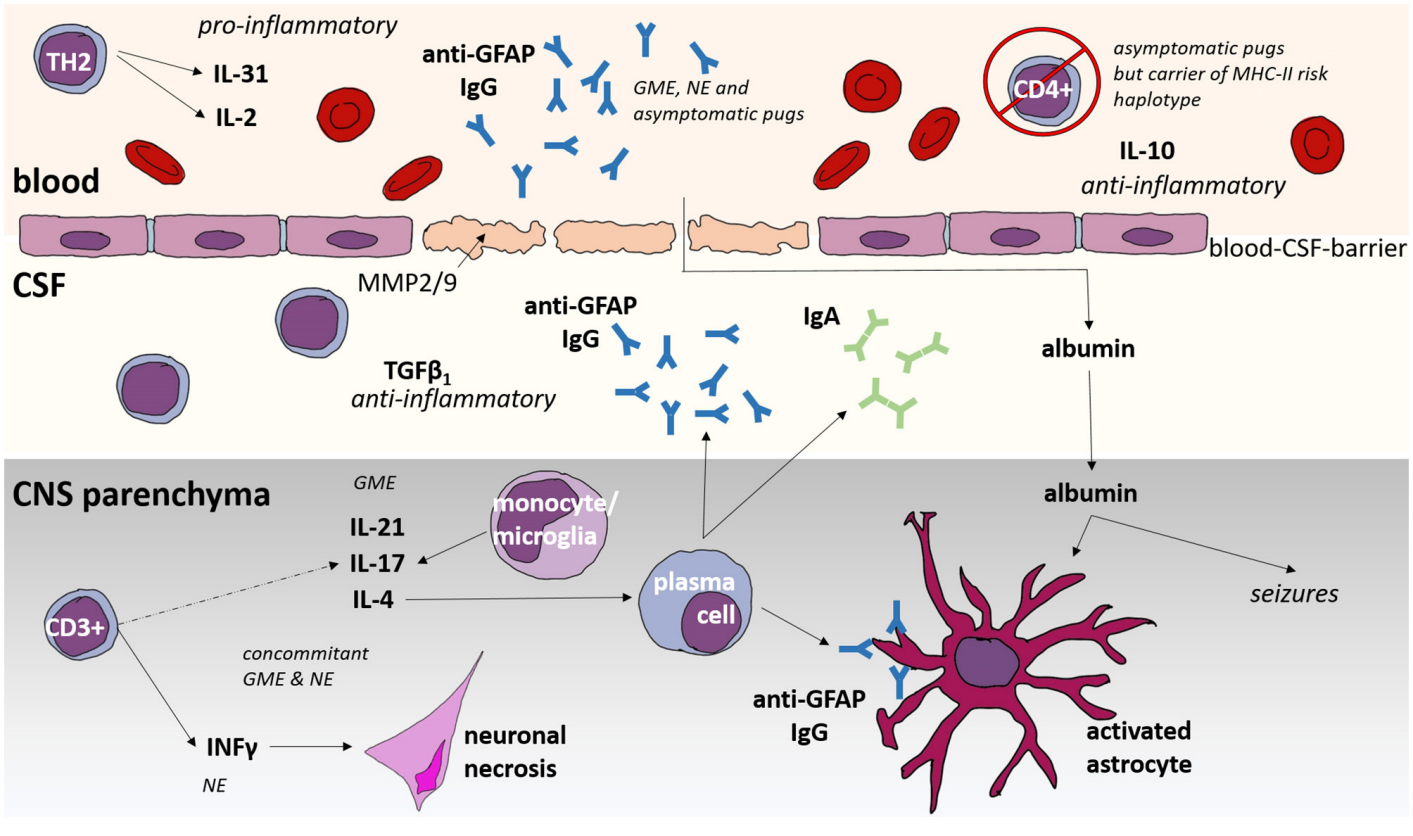
Immunological features of Meningoencephalitis of unknown origin (MUO) in dogs. GME, granulomatous meningoencephalomyelitis; NE, necrotizing encephalitis; CNS, central nervous system; CSF, cerebrospinal fluid; IL, interleukin; GFAP, glial fibrillary acid protein; Ig, immunoglobulin; MHC, Major Histocompatibility Complex; CD, cluster of differentiation; TGF, tumor growth factor; INF, interferon.

In dogs with MUO, we have identified significantly increased serum levels of IL-31 (Figure 1), a pro-inflammatory cytokine produced by Th2 cells (144). This finding was not observed across all cases: Dogs with elevated IL-31 that had histopathological phenotyping, particularly suffered from GME. Conversely, dogs with infectious meningoencephalitis did not demonstrate elevated IL-31 levels (144).

IL-31 plays a significant role in autoimmune diseases, particularly in human MS, where increased serum levels are prevalent (145). Treated patients with MS displayed a noteworthy reduction in IL-31 serum levels (145). Therefore, IL-31 warrants consideration as a potential prognostic marker for therapy when assessing the progression of MS. Due to the retrospective nature of our IL-31 study and the limited case number, evaluating whether dogs with MUO under treatment exhibit normalized IL-31 levels was not feasible (144).

Exploring the correlation between IL-31 levels and clinical signs, as well as the development of clinical signs depending on IL-31 levels, would be highly informative for future investigations. Additionally, pharmacological blockage of IL-31 might be a new therapeutic strategy to investigate. Lokivetmab is a monoclonal antibody against canine IL-31 and is currently approved for the treatment of itching in atopic dermatitis in dogs (146). It decreases clinical signs of pruritus within 3 h, and the effect of a subcutaneous injection lasts 28–48 days depending on the dosage (146). Long-term studies on healthy dogs showed no side effects beyond those of an unspecific reaction to any subcutaneous injection (147). Therefore, investigation into the clinical effectiveness of Lokivetmab against MUO could be worthwhile.

### 9.2 Blood-brain-barrier in MUO

The blood- and CSF-brain-barrier (BBB) serves as a highly regulated interface separating the CNS from the peripheral circulation and controlling the exchange of molecules to maintain CNS homeostasis (148). Comprising endothelial cells, pericytes, and astrocytes, the BBB relies on the pivotal role of astrocytes in its formation and maintenance by providing secreted factors that lead to the formation of strong tight junctions (148).

In neurological diseases, the BBB undergoes changes caused by phenotypical alterations in astrocytes amongst others, leading to increased permeability (149–151). This breach allows the extravasation of leukocytes, red blood cells, and plasma proteins into the CNS, as observed in murine experimental autoimmune encephalomyelitis, human MS, and canine MUO (107, 127, 152–155).

In cases of MUO, the BBB is compromised in the majority of dogs (156). Despite damage to astrocytes, the upregulation of MMP-2 contributes to the disruption of the blood-brain barrier (157). Glucocorticosteroid treatment proves effective in restoring the integrity of the blood-brain barrier by inducing the production of MMP inhibitors in SRMA (157) and this might also be the case in MUO.

Dogs with MUO show high levels of albumin in CSF and a high albumin cerebrospinal fluid/serum-quotient (QAlb) (158). As albumin is mostly produced extrathecally by the liver, it may serve as a marker for BBB damage (159).

The Reibergram, utilizing the serum:CSF ratio of albumin and correlating it with the serum:CSF ratio of biomarkers, provides a valuable tool for assessing the integrity of the BBB and determining whether a molecule was produced intrathecally or extrathecally (160). The hyperbolic curve QLim(IgA) = 0.13√((QAlb)^2^ + 11.9^*^10^−6^) −1.01^*^10^−3^ describes the upper reference value of the IgA serum:CSF ratio in correlation to the severity of BBB dysfunction (158). An autofill Excel spreadsheet for easy calculation and graphical evaluation of IgA and albumin ratios is available as supplemental data on the paper's journal homepage (158): https://onlinelibrary.wiley.com/action/downloadSupplement?doi=10.1111%2Fjvim.16601&file=jvim16601-sup-0001-Supinfo.zip.

The extravasation of albumin, in particular, can trigger the expression of proinflammatory cytokines, affecting the ability of astrocytes to maintain electrolyte homeostasis (161–164). This scenario may render neurons more susceptible to glutamate excitotoxicity, potentially causing seizures and exacerbate neuroinflammation (161, 165). Furthermore, albumin induces the production of CX3CL1, a chemokine that attracts CD4+ cells (163, 166).

Immunohistochemically activated astrocytes are visible in all subtypes of MUO (107, 167). In serum of Pug dogs with NME, GFAP is detectable in increased amounts (168) and anti-GFAP antibodies can be found in CSF and serum (129, 140, 141). Anti-GFAP antibodies can also be detected in asymptomatic Pug dogs (129), which might imply that the BBB is compromised before clinical signs become evident, allowing contact between self-reactive B cells and the brain, leading to the production of antibodies against astrocyte components. It is conceivable that autoimmunity has already started in these dogs, but anti-inflammatory mechanisms may be preventing the outbreak of NME, thereby maintaining their asymptomatic status.

If activation and destruction of astrocytes and BBB function are the hen or egg in MUO etiopathogenesis remains unclear at the moment. But it is almost evident that the combination of BBB damage, extravasated albumin, and impaired astrocyte function might be a major self-perpetuating vicious circle (Figure 1), which might be one of the major key points to be addressed in the future. Interrupting this circle is also one of the mechanisms of action of prednisolone: Glucocorticosteroid treatment proves effective in restoring the integrity of the blood-brain barrier by inducing the production of MMP inhibitors (157). Additionally, Telmisartan was reported in dogs with idiopathic epilepsy to possibly restore potential BBB damage (169) and could therefore be considered as future treatment option in MUO.

## 10 Further investigations: requirements and opportunities

Present treatment modalities, heavily reliant on long-term glucocorticosteroid application, may result in iatrogenic hyperadrenocorticism, adversely impacting the QoL of the pets and their owners (13, 47, 48). Attempts to decrease corticosteroid side effects through rapid dose tapering frequently lead to disease recurrence, necessitating additional immunomodulatory drugs (40, 57, 170). However, treatment efficacy remains suboptimal, marked by frequent relapses or insufficient clinical improvement (9).

A deeper understanding of the etiology of MUO is imperative for advancing therapeutic strategies. Critical to this advancement are multicenter studies aimed at unraveling the etiology of inflammatory CNS diseases, coupled with double-blinded multicenter treatment studies. Multicenter studies, by increasing the number of animals involved, allow for more precise examination of individual subgroups and factors such as differences in clinical signs, signalment, and epidemiology can be evaluated. Additionally, large numbers of animals enable the specific examination of homogenous groups with distinct features, such as different breed and age. A consensus on minimal diagnostic criteria is crucial, making multicenter retrospective studies challenging. Clear diagnostic criteria, including breed and age, preferably including biomarkers, are essential for multicentric studies.

## 11 Suggested polythetic approach for MUO spectrum

**Figure 2:**
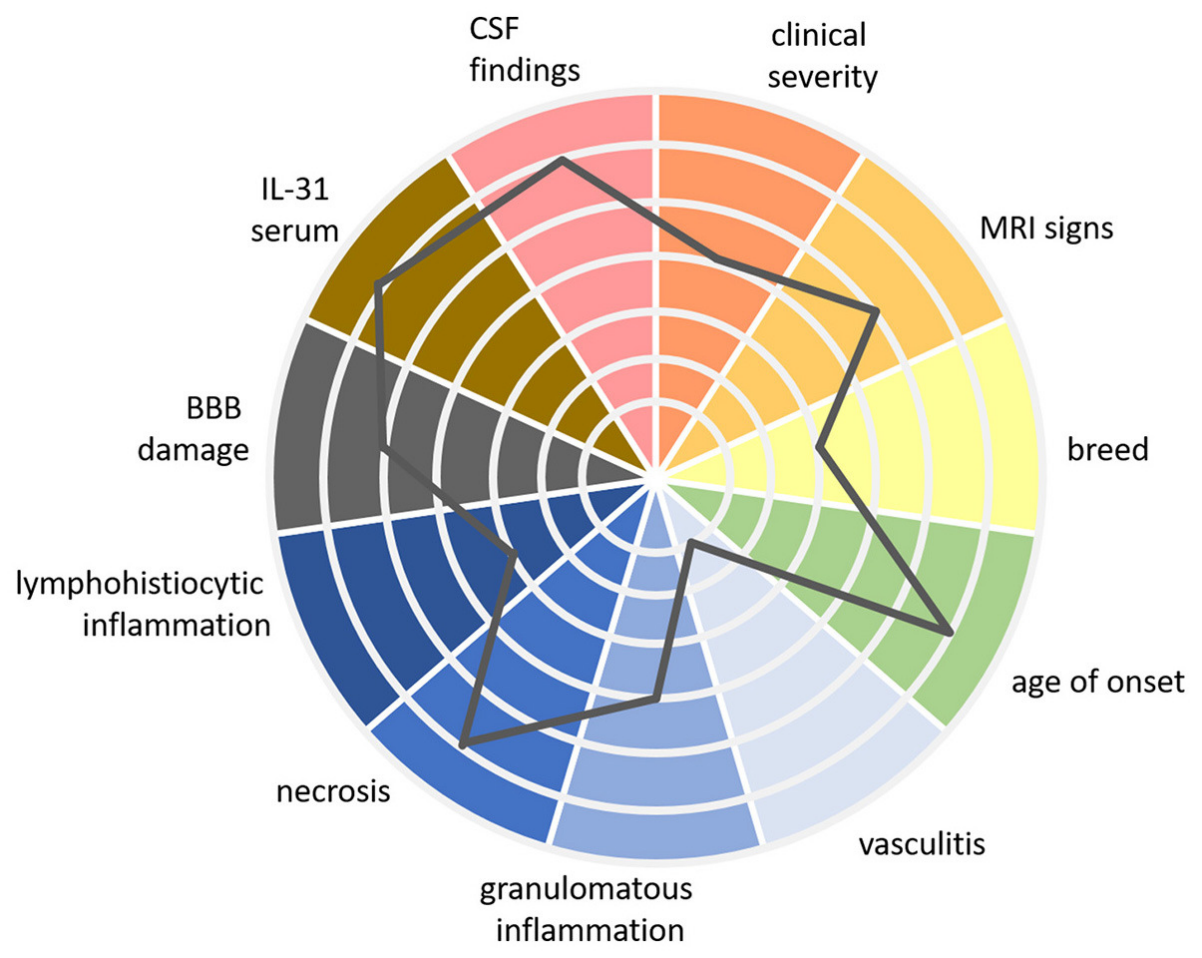
Patient specific MUO spectrum using a multidimensional polythetic approach. In this figure, we present a multidimensional, polythetic approach to characterizing patients within the MUO spectrum. Rather than proposing a rigid “classification” in the traditional sense—where patients are placed into well-defined, comparable classes—this approach recognizes the complexity and variability of MUO presentations. It emphasizes personalized assessment by considering multiple clinical and laboratory parameters, with each patient's profile represented as a unique combination of these variables. The gray line in the figure represents the findings for one individual patient, plotted across various axes to show the relative strength of each parameter. This figure is a proposed visual example based on current knowledge, intended to illustrate how different factors can be integrated into a personalized approach. Further research is required to refine the specific values and to better understand the clinical relevance of each marker within this model. While this polythetic approach does not yet define strict classes, it moves toward a framework that could, with additional evidence, lead to a more nuanced understanding of immune-mediated diseases like MUO. MUO, meningoencephalitis of unknown origin; CSF, cerebrospinal fluid; MRI, magnetic resonance imaging; IL, interleukin; BBB, blood-brain-barrier.

For the future, a patient-specific therapy would be desirable. Tailoring treatment to the individual patient's needs could enhance the effectiveness of interventions while minimizing potential side effects. Therefore, it would be beneficial to establish a multifaceted classification system including more than histopathological findings alone. The author suggests conceptualizing the multidimensional spectrum of MUO with a polythetic approach (Figure 2).

“Polythetic” means that members of a group share a subset of characteristics but not necessarily all of them (171, 172). In other words, there is a certain degree of variability in the traits exhibited by the individual within a particular category. This could mean for MUO, that it might be characterized by multiple clinical signs, examination findings, and biomarkers, but not every listed sign or finding is required to diagnose the condition in a particular animal. It might include age of onset of clinical signs, breed, quality and quantity of clinical signs, MRI and CSF findings, severity of BBB damage, serum IL-31 levels and different histopathological features.

For example, different therapeutic options could be tailored to individual patients based on their specific manifestations across various dimensions within the polythetic approach. For example, dogs with a mostly necrotizing variant of MUO might benefit from treatments targeting IFN-γ inhibition, while those with elevated IL-31 levels could respond better to anti-IL-31 antibody therapies. Similarly, dogs with impaired BBB function may benefit from treatments aimed at restoring BBB integrity, which need to be developed in the future.

Furthermore, the polythetic approach may enable us to classify MUO subtypes more precisely than current histopathological methods, helping to develop for example more specific diagnostic biomarkers. This approach could help reveal relationships and patterns that are not apparent with our current understanding, leading to a better understanding of the etiopathogenesis and more targeted and effective treatments.

Using a multidimensional polythetic approach, patient-specific etiopathogenesis enables neurologists to integrate innovative, pathophysiologically based treatments with objective tests—such as stratification biomarkers—to anticipate the potential benefits of distinct treatments for individual cases. This approach allows for tailoring treatment schedules to the patient's specific needs at a given time, reducing the need for long-term prednisolone therapy, minimizing side effects, and improving both treatment efficacy and quality of life.

## 12 Conclusion

This review lays the basics for forthcoming therapeutic trials, aiming to advance the development of minimally side-effect therapies for patients suffering from MUO.

A polythetic approach that represents the multidimensional spectrum of MUO could adequately address patient-specific needs and potentially decrease adverse effects while improving quality of life. To attain a comprehensive understanding of etiopathogenesis, conducting larger multicenter studies are necessary to recruit an adequate number of patients. For effective multicenter studies, it is crucial to streamline examination procedures across clinics and subsequent analysis.

This review underscores the potential for fostering renewed consensus on diagnostic and classification practices within the diverse spectrum of autoimmune CNS diseases in cats and dogs, serving as a catalyst for prospective treatment studies and encouraging collaborative agreement among researchers and clinicians.

## References

[1] Schwab S Herden C Seeliger F Papaioannou N Psalla D Polizopulou Z. Non-suppurative meningoencephalitis of unknown origin in cats and dogs: an immunohistochemical study. J Comp Pathol. (2007) 136:96–110. 10.1016/j.jcpa.2006.11.006 17275833 PMC7126569

[2] Talarico LR Schatzberg SJ. Idiopathic granulomatous and necrotising inflammatory disorders of the canine central nervous system: a review and future perspectives. J Small Anim Pract. (2010) 51:138–49. 10.1111/j.1748-5827.2009.00823.x 19814766

[3] Cornelis I Van Ham L Gielen I Decker SD Bhatti SFM. Clinical presentation, diagnostic findings, prognostic factors, treatment and outcome in dogs with meningoencephalomyelitis of unknown origin: a review. Vet J. (2019) 244:37–44. 10.1016/j.tvjl.2018.12.007 30825893

[4] Granger N Smith PM Jeffery ND. Clinical findings and treatment of non-infectious meningoencephalomyelitis in dogs: a systematic review of 457 published cases from 1962 to 2008. Vet J. (2010) 184:290–7. 10.1016/j.tvjl.2009.03.031 19410487

[5] Goncalves R Decker SD Walmsley G Butterfield S Maddox TW. Inflammatory disease affecting the central nervous system in dogs: a retrospective study in England (2010-2019). Front Vet Sci. (2021) 8:819945. 10.3389/fvets.2021.819945 35155652 PMC8829331

[6] Tipold A Fatzer R Jaggy A Zurbriggen A Vandevelde M. Necrotizing encephalitis in Yorkshire terriers. J Small Anim Pract. (1993) 34:623–28. 10.1111/j.1748-5827.1993.tb02598.x

[7] Hoff EJ Vandevelde M. Case Report: Necrotizing vasculitis in the central nervous systems of two dogs. Vet Pathol. (1981) 18:219–23. 10.1177/030098588101800209 7467081

[8] Schatzberg SJ Haley NJ Barr SC Lahunta A Sharp NJH. Polymerase chain reaction screening for DNA viruses in paraffin-embedded brains from dogs with necrotizing meningoencephalitis, necrotizing leukoencephalitis, and granulomatous meningoencephalitis. J Vet Intern Med. (2005) 19:553–9. 10.1111/j.1939-1676.2005.tb02726.x 16095173

[9] Jeffery N Granger N. New insights into the treatment of meningoencephalomyelitis of unknown origin since 2009: a review of 671 cases. Front Vet Sci. (2023) 10:1114798. 10.3389/fvets.2023.1114798 37008358 PMC10050685

[10] Beasley MJ Shores A. Perspectives on pharmacologic strategies in the management of meningoencephalomyelitis of unknown origin in dogs. Front Vet Sci. (2023) 10:1167002. 10.3389/fvets.2023.1167002 37234070 PMC10205981

[11] Jones BS Liebel FX Fadda A Martin S Lawn R Lazzerini K. Corticosteroid monotherapy versus combined cytarabine continuous rate infusion and corticosteroid therapy in dogs with meningoencephalitis of unknown origin: a blinded, randomized, controlled trial. J Vet Intern Med. (2024) 38:1618–25. 10.1111/jvim.17088 38700360 PMC11099798

[12] Hampel H Caraci F Cuello AC Caruso G Nisticò R Corbo M. A path toward precision medicine for neuroinflammatory mechanisms in Alzheimer's disease. Front Immunol. (2020) 11:456. 10.3389/fimmu.2020.00456 32296418 PMC7137904

[13] Viviano KR. Glucocorticoids, cyclosporine, azathioprine, chlorambucil, and mycophenolate in dogs and cats clinical uses, pharmacology, and side effects. Vet Clin North Am Small Anim Pract. (2022) 52:797–817. 10.1016/j.cvsm.2022.01.009 35379498

[14] Andersen-Ranberg E Berendt M Gredal H. Biomarkers of non-infectious inflammatory CNS diseases in dogs - Where are we now? Part I: Meningoencephalitis of unknown origin. Vet J. (2021) 273:105678. 10.1016/j.tvjl.2021.105678 34148601

[15] Choi Y Harcourt-Brown T Ives E Lopes B Alves LC Cardy T. Investigation on canine MUO at veterinary referral care in England (2017-2021): a multicentric retrospective study. In: 34rd European Society of Veterinary Neurology (ESVN)-European College of Veterinary Neurology (ECVN) Symposium, Venice, Italy (2023). p. 53.

[16] Diangelo L Cohen-Gadol A Heng HG Miller MA Hague DW Rossmeisl JH. Glioma mimics: magnetic resonance imaging characteristics of granulomas in dogs. Front Vet Sci. (2019) 6:286. 10.3389/fvets.2019.00286 31555671 PMC6722480

[17] Koestner A Zeman Z. Primary reticuloses of the central nervous system in dogs. Am J Vet Res. (1962) 23:381–93. 14457741

[18] Cameron AM Conroy JD. Rabies-like neuronal inclusions associated with a neoplastic reticulosis in a dog. Vet Path. (1974) 11:29–37. 10.1177/030098587401100104 4139800

[19] Braund KG Vandevelde M Walker TL Redding RW. Granulomatous meningoencephalomyelitis in six dogs. J Am Vet Med Assoc. (1978) 172:1195–200. 659301

[20] Vandevelde M Kristensen B Greene CE. Primary reticulosis of the central nervous system in the dog. Vet Pathol. (1978) 15:673–5. 10.1177/030098587801500512 716161

[21] Cordy DR. Canine granulomatous meningoencephalomyelitis. Vet Pathol. (1979) 16:325–33. 10.1177/030098587901600306 442463

[22] Russo ME. Primary reticulosis of the central nervous system in dogs. J Am Vet Med Assoc. (1979) 174:492–500. 447580

[23] Vandevelde M Fatzer R Fankhauser R. Imniunohistological studies on primary reticulosis of the canine brain. Vet Palhol. (1981) 18:577–88. 10.1177/030098588101800502 6895132

[24] O'Neill EJ Merrett D Jones B. Granulomatous meningoencephalomyelitis in dogs: a review. Ir Vet J. (2005) 58:87–92. 10.1186/2046-0481-58-2-86 21851667 PMC3113901

[25] Barber RM Porter BF Li Q May M Claiborne MK Allison AB. Broadly reactive polymerase chain reaction for pathogen detection in canine granulomatous meningoencephalomyelitis and necrotizing meningoencephalitis. J Vet Intern Med. (2012) 26:962–8. 10.1111/j.1939-1676.2012.00954.x 22686439 PMC7166683

[26] Hoon-Hanks LL McGrath S Tyler KL Owen C Stenglein MD. Metagenomic investigation of idiopathic meningoencephalomyelitis in dogs. J Vet Intern Med. (2018) 32:324–30. 10.1111/jvim.14877 29197179 PMC5787199

[27] Vandevelde M Boring JG Hoff EJ Gingerich DA. The effect of levamisole on the canine central nervous system. J Neuropathol Exp Neurol. (1978) 37:165–73. 10.1097/00005072-197803000-00005 632846

[28] Kipar A Baumgartner W Vogl C Gaedke K Wellman M. Immunohistochemical characterization of inflammatory cells in brains of dogs with granulomatous meningoencephalitis. Vet Pathol. (1998) 35:43–52. 10.1177/030098589803500104 9545134

[29] Cordy DR Holliday TA. A necrotizing meningoencephalitis of pug dogs. Vet Pathol. (1989) 26:191–4. 10.1177/030098588902600301 2763409

[30] Lezmi S Toussaint Y Prata D Lejeune T Ferreira-Neves P Rakotovao F. Severe necrotizing encephalitis in a Yorkshire terrier: topographic and immunohistochemical study. J Vet Med A Physiol Pathol Clin Med. (2007) 54:186–90. 10.1111/j.1439-0442.2007.00925.x 17493164

[31] Speciale J Van Winkle TJ Steinberg SA Wortman JA. Computed tomography in the diagnosis of focal granulomatous meningoencephalitis: retrospective evaluation of th ree gases. J Am Vet Med Assoc. (1992) 28:327–32.

[32] Tanaka M Inoue A Yamamoto K Tamahara S Matsuki N. Transglutaminase 2: a novel autoantigen in canine idiopathic central nervous system inflammatory diseases. J Vet Med Sci. (2012) 74:733–7. 10.1292/jvms.11-0507 22230982

[33] Cornelis I Volk HA Decker SD. Clinical presentation, diagnostic findings and long-term survival in large breed dogs with meningoencephalitis of unknown aetiology. Vet Rec. (2016) 179:147. 10.1136/vr.103640 27165997

[34] Elbert JA Yau W Rissi DR. Neuroinflammatory diseases of the central nervous system of dogs: a retrospective study of 207 cases (2008-2019). Can Vet J. (2022) 63:178–86. 35110776 PMC8759338

[35] Zarfoss M Schatzberg S Venator K Cutter-Schatzberg K Cuddon P Pintar J. Combined cytosine arabinoside and prednisone therapy for meningoencephalitis of unknown aetiology in 10 dogs. J Small Anim Pract. (2006) 47:588–95. 10.1111/j.1748-5827.2006.00172.x 17004951

[36] Coates JR Barone G Dewey CW Vitale CL Holloway-Azene NM Sessions JK. Procarbazine as adjunctive therapy for treatment of dogs with presumptive antemortem diagnosis of granulomatous meningoencephalomyelitis: 21 cases (1998–2004). J Vet Intern Med. (2008) 21:100–6. 10.1111/j.1939-1676.2007.tb02934.x 17338156

[37] Smith PM Stalin CE Shaw D Granger N Jeffery ND. Comparison of two regimens for the treatment of meningoencephalomyelitis of unknown etiology. J Vet Intern Med. (2009) 23:520–6. 10.1111/j.1939-1676.2009.0299.x 19645837

[38] Wong MA Hopkins AL Meeks JC Clarke JD. Evaluation of treatment with a combination of azathioprine and prednisone in dogs with meningoencephalomyelitis of undetermined etiology: 40 cases (2000–2007). J Am Vet Med Assoc. (2010) 237:929–935. 10.2460/javma.237.8.929 20946080

[39] Flegel T Boettcher IC Matiasek K Oevermann A Doherr MG Oechtering G. Comparison of oral administration of lomustine and prednisolone or prednisolone alone as treatment for granulomatous meningoencephalomyelitis or necrotizing encephalitis in dogs. J Am Vet Med Assoc. (2011) 238:337–45. 10.2460/javma.238.3.337 21281217

[40] Lowrie M Smith PM Garosi L. Meningoencephalitis of unknown origin: investigation of prognostic factors and outcome using a standard treatment protocol. Vet Rec. (2013) 172:527. 10.1136/vr.101431 23462382

[41] Beckmann K Carrera I Steffen F Golini L Kircher PR Schneider U. A newly designed radiation therapy protocol in combination with prednisolone as treatment for meningoencephalitis of unknown origin in dogs: a prospective pilot study introducing magnetic resonance spectroscopy as monitor tool. Acta Vet Scand. (2015) 57:4. 10.1186/s13028-015-0093-3 25637270 PMC4316757

[42] Mercier M Barnes Heller HL. Efficacy of glucocorticoid monotherapy for treatment of canine meningoencephalomyelitis of unknown etiology: a prospective study in 16 dogs. Vet Med Sci. (2015) 1:16–22. 10.1002/vms3.4 29067170 PMC5645807

[43] Barnoon I Shamir MH Aroch I Bdolah-Abram T Srugo I Konstantin L. Retrospective evaluation of combined mycophenolate mofetil and prednisone treatment for meningoencephalomyelitis of unknown etiology in dogs: 25 cases (2005-2011). J Vet Emerg Crit Care. (2016) 26:116–24. 10.1111/vec.12399 26458162

[44] Woolcock AD Wang A Haley A Kent M Creevy KE Platt SR. Treatment of canine meningoencephalomyelitis of unknown aetiology with mycophenolate mofetil and corticosteroids: 25 cases (2007-2012). Vet Med Sci. (2016) 2:125–35. 10.1002/vms3.22 29067186 PMC5645855

[45] Pausova TK Tomek A Srenk P Belaskova S. Clinical presentation, diagnostic findings, and long-term survival time in 182 dogs with meningoencephalitis of unknown origin from central europe that were administered glucocorticosteroid monotherapy. Top Companion Anim Med. (2021) 44:100539. 10.1016/j.tcam.2021.100539 33964477

[46] Barber R Downey Koos L. Treatment with cytarabine at initiation of therapy with cyclosporine and glucocorticoids for dogs with meningoencephalomyelitis of unknown origin is not associated with improved outcomes. Front Vet Sci. (2022) 9:925774. 10.3389/fvets.2022.925774 35754543 PMC9226772

[47] Heidemann PL Erhald B Koch BC Gredal H. Investigation of side effects to treatment and cause of death in 63 Scandinavian dogs suffering from meningoencephalitis of unknown origin: a retrospective study. Acta Vet Scand. (2023) 65:46. 10.1186/s13028-023-00709-7 37858113 PMC10588026

[48] Sri-Jayantha LS Doornink MT Urie BK. Increased risk of select glucocorticoid adverse events in dogs of higher body weight. Can Vet J. (2022) 63:32–8. 34975165 PMC8682939

[49] Lawn RW Harcourt-Brown TR. Risk factors for early death or euthanasia within 100 days of diagnosis in dogs with meningoencephalitis of unknown origin. Vet J. (2022) 287:105884. 10.1016/j.tvjl.2022.105884 35987308

[50] Smith SM Westermeyer HD Mariani CL Gilger BC Davidson MG. Optic neuritis in dogs: 96 cases (1983-2016). Vet Ophthalmol. (2018) 21:442–51. 10.1111/vop.12528 29251394

[51] Maehara T Shimada A Morita T Sawashima Y Sawashima K. Distribution of the inflammatory lesions in the central nervous system of dogs affected with disseminated and ocular form of granulomatous meningoencephalomyelitis. J Vet Med Sci. (2009) 71:509–12. 10.1292/jvms.71.509 19420859

[52] Higgins RJ Dickinson PJ Kube SA Moore PF Couto SS Vernau KM. Necrotizing Meningoencephalitis in Five Chihuahua Dogs. Vet Pathol. (2008) 45:336–46. 10.1354/vp.45-3-336 18487490

[53] Levine JM Fosgate GT Porter B Schatzberg SJ Greer K. Epidemiology of necrotizing meningoencephalitis in Pug dogs. J Vet Intern Med. (2008) 22:961–8. 10.1111/j.1939-1676.2008.0137.x 18647157 PMC7166975

[54] Cornelis I Volk HA Van Ham L Decker SDe. Clinical presentation, diagnostic findings and outcome in dogs diagnosed withpresumptive spinal-only meningoen-cephalomyelitis of unknown origin. J Small Anim Pract. (2017) 58:174–82. 10.1111/jsap.12622 28267222 PMC7166691

[55] Munana KR Luttgen PJ. Prognostic factors for dogs with granulomatous meningoencephalomyelitis: 42 cases (1982-1996). J Am Vet Med Assoc. (1998) 212:1902–6. 10.2460/javma.1998.212.12.1902 9638190

[56] Gonçalves R Maddox TW Phillipps S Nagendran A Cooper C Orlandi R. Development of a reliable clinical assessment tool for meningoencephalitis in dogs: the neurodisability scale. J Vet Intern Med. (2023) 37:1111–8. 10.1111/jvim.16717 37092590 PMC10229334

[57] Brewińska L Banasik A Czopowicz M Płonek M Giza E Czerwik A. Usefulness of neurological assessment scales in prognosis of meningoencephalitis of unknown origin in Yorkshire Terriers. PREPRINT (Version 1) published on Research Square (2024). 10.21203/rs.3.rs-4314467/v1 PMC11863485 40011993

[58] Cherubini GB Platt SR Anderson TJ Rusbridge C Lorenzo V Mantis P. Characteristics of magnetic resonance images of granulomatous meningoencephalomyelitis in 11 dogs. Vet Rec. (2006) 159:110–5. 10.1136/vr.159.4.110 16861389

[59] Flegel T. Breed-specific magnetic resonance imaging characteristics of necrotizing encephalitis in dogs. Front Vet Sci. (2017) 4:203. 10.3389/fvets.2017.00203 29255715 PMC5723069

[60] Wu CC Chang YP. Long-term outcomes and prognostic factors in dogs with meningoencephalitis of unknown origin and suspected necrotic lesions on magnetic resonance imaging: 37 cases (2007-2020). J Am Vet Med Assoc. (2024) 1:1–9. 10.2460/javma.24.03.0222 38901458

[61] Young BD Levine JM Fosgate GT Lahunta Ad Flegel T Matiasek K. Magnetic resonance imaging characteristics of necrotizing meningoencephalitis in Pug dogs. J Vet Intern Med. (2009) 23:527–35. 10.1111/j.1939-1676.2009.0306.x 19645838

[62] Cavalerie R Santos ACD Leonardi H Blond L Beurlet S Dumont R. C-reactive protein concentration has limited value in the diagnosis of meningoencephalitis of unknown origin in dogs. J Am Vet Med Assoc. (2024) 262:481–8. 10.2460/javma.23.11.0606 38266391

[63] Jones BS Harcourt-Brown T. Comparison of serum creatine kinase and aspartate aminotransferase activity in dogs with Neospora meningoencephalitis and noninfectious meningoencephalitis. J Vet Intern Med. (2022) 36:141–5. 10.1111/jvim.16334 34859908 PMC8783338

[64] Galer J Forward AK Hughes J Crawford AH Behr S Cherubini GB. Clinical features, treatment, and outcome of juvenile dogs with meningoencephalitis of unknown etiology. J Vet Intern Med. (2024) 38:2214–20. 10.1111/jvim.17126 38932495 PMC11256174

[65] Flegel T Oevermann A Oechtering G Matiasek K. Diagnostic yield and adverse effects of MRI-guided free-hand brain biopsies through a mini-burr hole in dogs with encephalitis. J Vet Intern Med. (2012) 26:969–76. 10.1111/j.1939-1676.2012.00961.x 22708694

[66] Cornelis I Volk HA Van Ham L Decker SDe. Prognostic factors for 1-week survival in dogs diagnosed with meningoencephalitis of unknown aetiology. Vet J. (2016) 214:91–5. 10.1016/j.tvjl.2016.05.008 27387733

[67] Oliphant BJ Barnes Heller HL White JM. Retrospective study evaluating associations between midline brain shift on magnetic resonance imaging and survival in dogs diagnosed with meningoencephalitis of unknown etiology. Vet Radiol Ultrasound. (2017) 58:38–43. 10.1111/vru.12434 27774741

[68] Gonçalves R Decker SD Walmsley G Maddox TW. Magnetic resonance imaging prognostic factors for survival and relapse in dogs with meningoencephalitis of unknown origin. Front Vet Sci. (2024) 11:1370882. 10.3389/fvets.2024.1370882 38482167 PMC10933066

[69] Ostrager A Bentley RT Lewis MJ Moore GE. Survival in dogs with meningoencephalomyelitis of unknown etiology with and without lesions detected by magnetic resonance imaging. J Vet Intern Med. (2024) 38:2204–13. 10.1111/jvim.17109 38804716 PMC11256124

[70] Negrin A Spencer S Cherubini GB. Feline meningoencephalomyelitis of unknown origin: a retrospective analysis of 16 cases. Can Vet J. (2017) 58:1073–80. 28966357 PMC5603942

[71] Bradshaw JM Pearson GR Gruffydd-Jones TJ. A retrospective study of 286 cases of neurological disorders of the cat. J Comp Pathol. (2004) 131:112–20. 10.1016/j.jcpa.2004.01.010 15276850 PMC7134559

[72] Rand JS Parent J Percy D Jacobs R. Clinical, cerebrospinal fluid, and histological data from twenty-seven cats with primary inflammatory disease of the central nervous system. Can Vet J. (1994) 35:103–10. 8069819 PMC1686724

[73] Singh M Foster DJ Child G Lamb WA. Inflammatory cerebrospinal fluid analysis in cats: Clinical diagnosis and outcome. J Feline Med Surg. (2005) 7:77–93. 10.1016/j.jfms.2004.07.001 15771944 PMC7128774

[74] Risio LD Brown R Tennant B Sparkes A Matiasek L Stefani Ad. Slowly progressive lymphohistiocytic meningoencephalomyelitis in 21 adult cats presenting with peculiar neurological signs. J Feline Med Surg. (2012) 14:250–6. 10.1177/1098612X11435460 22412162 PMC10822513

[75] Lundgren A-L. Feline non-suppurative meningoencephalomyelitis. A clinical and pathological study. J Comp Pathol. (1992) 107:411–25. 10.1016/0021-9975(92)90015-M 1291589 PMC7130315

[76] Nowotny N Weissenböck H. Description of feline nonsuppurative meningoencephalomyelitis (“staggering disease”) and studies of its etiology. J Clin Microbiol. (1995) 33:1668–9. 10.1128/jcm.33.6.1668-1669.1995 7650212 PMC228243

[77] Lundgren A-L Johannisson A Zimmermann W Bode L Rozell B Muluneh A. Neurological disease and encephalitis in cats experimentally infected with Borna disease virus. Acta Neuropathol. (1997) 93:391–401. 10.1007/s004010050630 9113204 PMC7086795

[78] Nishino Y Funaba M Fukushima R Mizutami T Kimura T Iizuka R. Borna disease virus infection in domestic cats: evaluation by RNA and antibody detection. J Vet Med Sci. (1999) 61:1167–70. 10.1292/jvms.61.1167 10563298

[79] Lundgren A-L Lindberg R Ludwig H Gosztonyi G. Immunoreactivity of the central nervous system in cats with a Borna disease-like meningoencephalomyelitis (staggering disease). Acta Neuropathol. (1995) 90:184–93. 10.1007/BF00294319 7484095 PMC7086677

[80] Matiasek K Pfaff F Weissenböck H Wylezich C Kolodziejek J Tengstrand S. Mystery of fatal ‘staggering disease' unravelled: novel rustrela virus causes severe meningoencephalomyelitis in domestic cats. Nat Commun. (2023) 14:624. 10.1038/s41467-023-36204-w 36739288 PMC9899117

[81] Nessler J Wohlsein P Junginger J Hansmann F Erath J Söbbeler F. Meningoencephalomyelitis of unknown origin in cats: a case series describing clinical and pathological findings. Front Vet Sci. (2020) 7:291. 10.3389/fvets.2020.00291 32671104 PMC7326087

[82] Nessler JN Jo WK Osterhaus ADME Ludlow M Tipold A. Canine meningoencephalitis of unknown origin- the search for infectious agents in the cerebrospinal fluid via deep sequencing. Front Vet Sci. (2021) 8:645517. 10.3389/fvets.2021.645517 34950723 PMC8688736

[83] Nippert S Rubbenstroth D Geers JA Ebinger A Hoffmann D Breithaupt A. Continuous presence of genetically diverse rustrela virus lineages in yellow-necked field mouse reservoir populations in northeastern Germany. Virus Evol. (2023) 9:vead048. 10.1093/ve/vead048 37744713 PMC10516363

[84] Le Roi MD Puff C Wohlsein P Pfaff F Beer M Baumgärtner W. Rustrela virus as putative cause of nonsuppurative meningoencephalitis in lions. Emerg Infect Dis. (2023) 29:1042. 10.3201/eid2905.230172 37081716 PMC10124629

[85] Voss A Schlieben P Gerst S Wylezich C Pfaff F Langner C. Rustrela virus infection - An emerging neuropathogen of red-necked wallabies (*Macropus rufogriseus*). Transbound Emerg Dis. (2022) 69:4016–21. 10.1111/tbed.14708 36135593

[86] Weiss V Weidinger P Matt J Weissenbacher-Lang C Nowotny N Weissenbock H. Rustrela virus-associated encephalomyelitis (‘staggering disease') in cats from eastern Austria, 1994-2016. Viruses. (2023) 15:1621. 10.3390/v15081621 37631964 PMC10458416

[87] McNamara J Drake J Wiseman S Wright I. Survey of European pet owners quantifying endoparasitic infection risk and implications for deworming recommendations. Parasit Vectors. (2018) 11:571. 10.1186/s13071-018-3149-1 30382932 PMC6211546

[88] Bach J-F. Revisiting the hygiene hypothesis in the context of autoimmunity. Front Immunol. (2021) 11:615192. 10.3389/fimmu.2020.615192 33584703 PMC7876226

[89] Hemida MBM Vuori KA Borgstrom NC Moore R Rosendahl S Anturaniemi J. Early life programming by diet can play a role in risk reduction of otitis in dogs. Front Vet Sci. (2023) 10:1186131. 10.3389/fvets.2023.1186131 38026629 PMC10657834

[90] Junginger J Raue K Wolf K Janecek E Stein VM Tipold A. Zoonotic intestinal helminths interact with the canine immune system by modulating T cell responses and preventing dendritic cell maturation. Sci Rep. (2017) 7:10310. 10.1038/s41598-017-10677-4 28871165 PMC5583179

[91] Roussel C Drake J Ariza JM. French national survey of dog and cat owners on the deworming behaviour and lifestyle of pets associated with the risk of endoparasites. Parasit Vectors. (2019) 12:480. 10.1186/s13071-019-3712-4 31610795 PMC6792328

[92] Sabel CE Pearson JF Mason DF Willoughby E Abernethy DA Taylor BV. The latitude gradient for multiple sclerosis prevalence is established in the early life course. Brain. (2021) 144:2038–46. 10.1093/brain/awab104 33704407

[93] Ismailova K Poudel P Parlesak A Frederiksen P Heitmann BL. Vitamin D in early life and later risk of multiple sclerosis—A systematic review, meta-analysis. PLoS ONE. (2019) 14:e0221645. 10.1371/journal.pone.0221645 31454391 PMC6711523

[94] How K Hazewinkel H Mol J. Photosynthesis of vitamin d in the skin of dogs, cats, and rats. Vet Q. (1995) 17:29–29. 10.1080/01652176.1995.9694579 7571299

[95] Laing C Malik R Wigney D Fraser D. Seasonal vitamin D status of Greyhounds in Sydney. Aust Vet J. (1999) 77:35–8. 10.1111/j.1751-0813.1999.tb12425.x 10028392

[96] Anfinsen KP Berendt M Liste FJ Haagensen TR Indrebo A Lingaas F. A retrospective epidemiological study of clinical signs and familial predisposition associated with aseptic meningitis in the Norwegian population of Nova Scotia duck tolling retrievers born 1994–2003. Can J Vet Res. (2008) 72:350. 18783024 PMC2442678

[97] Hartcourt RA. Polyarteritis in a colony of beagles. Vet Rec. (1978) 102:519–22. 10.1136/vr.102.24.519 675999

[98] Windsor R Stewart S Schmidt J Mosqueda M Piras I Keller SM. A potential early clinical phenotype of necrotizing meningoencephalitis in genetically at-risk pug dogs. J Vet Intern Med. (2022) 36:1382–9. 10.1111/jvim.16444 35621070 PMC9308433

[99] Schrauwen I Barber RM Schatzberg SJ Siniard AL Corneveaux JJ Porter BF. Identification of novel genetic risk loci in maltese dogs with necrotizing meningoencephalitis and evidence of a shared genetic risk across toy dog breeds. PLoS ONE. (2014) 9:e112755. 10.1371/journal.pone.0112755 25393235 PMC4231098

[100] Kajin F Schuwerk L Beineke A Volk HA Meyerhoff N Nessler J. Teach an old dog new tricks: Meningoencephalitis of unknown origin (MUO) in Australian shepherd dogs. Vet Rec Case Rep. (2023) 11:e589. 10.1002/vrc2.589

[101] Greer KA Schatzberg SJ Porter BF Jones KA Famula TR Murphy KE. Heritability and transmission analysis of necrotizing meningoencephalitis in the Pug. Res Vet Sci. (2009) 86:438–42. 10.1016/j.rvsc.2008.10.002 19014875

[102] Greer KA Wong AK Liu H Famula TR Pedersen NC Ruhe A. Necrotizing meningoencephalitis of Pug dogs associates with dog leukocyte antigen class II and resembles acute variant forms of multiple sclerosis. Tissue Antigens. (2010) 76:110–8. 10.1111/j.1399-0039.2010.01484.x 20403140

[103] van Renen J Kehl A Buhmann G Matiasek LA Zablotski Y Fischer A. Allele frequency of a genetic risk variant for necrotizing meningoencephalitis in pug dogs from Europe and association with the clinical phenotype. Front Vet Sci. (2024) 11:1407288. 10.3389/fvets.2024.1407288 38840637 PMC11150678

[104] Oshima A Ito D Katakura F Miyamae J Okano M Nakazawa M. Dog leukocyte antigen class II alleles and haplotypes associated with meningoencephalomyelitis of unknown origin in Chihuahuas. J Vet Med Sci. (2023) 85:62–70. 10.1292/jvms.22-0116 36418080 PMC9887217

[105] Ishina IA Zakharova MY Kurbatskaia IN Mamedov AE Belogurov AA Gabibov AG. MHC class II presentation in autoimmunity. Cells. (2023) 12:314. 10.3390/cells12020314 36672249 PMC9856717

[106] Massey J Rothwell S Rusbridge C Tauro A Addicott D Chinoy H. Association of an MHC class II haplotype with increased risk of polymyositis in Hungarian Vizsla dogs. PLoS ONE. (2013) 8:e56490. 10.1371/journal.pone.0056490 23457575 PMC3572995

[107] Uchida K Park E Tsuboi M Chambers JK Nakayama H. Pathological and immunological features of canine necrotising meningoencephalitis and granulomatous meningoencephalitis. Vet J. (2016) 213:72–7. 10.1016/j.tvjl.2016.05.002 27240919

[108] Park ES Uchida K Nakayama H. Comprehensive immunohistochemical studies on canine necrotizing meningoencephalitis (NME), necrotizing leukoencephalitis (NLE), and granulomatous meningoencephalomyelitis (GME). Vet Pathol. (2012) 49:682–92. 10.1177/0300985811429311 22262353

[109] Oberheim NA Goldman SA Nedergaard M. Heterogeneity of astrocytic form and function. Methods Mol Biol. (2012) 814:23–45. 10.1007/978-1-61779-452-0_3 22144298 PMC3506190

[110] Nessler JN Oevermann A Schawacht M Gerhauser I Spitzbarth I Bittermann S. Concomitant necrotizing encephalitis and granulomatous meningoencephalitis in four toy breed dogs. Front Vet Sci. (2022) 9:957285. 10.3389/fvets.2022.957285 36118343 PMC9477003

[111] Zdora I Raue J Sobbeler F Tipold A Baumgartner W Nessler JN. Case report: Lympho-histiocytic meningoencephalitis with central nervous system vasculitis of unknown origin in three dogs. Front Vet Sci. (2022) 9:944867. 10.3389/fvets.2022.944867 36090171 PMC9449415

[112] Robinson K. Transcriptomic evaluation of granulomatous and nectrotizing meningoencephalitis of dogs. University of Georgia, Athens, Georgia (2020). p. 80.

[113] Schaudien D Schwab S Linke S Seeliger F Pauli G Baumgärtner W. Lack of detectable West Nile virus RNA in brains and kidneys of dogs and cats with immunohistological precipitates using virus-specific antibodies. Vet Microbiol. (2008) 132:171–6. 10.1016/j.vetmic.2008.05.007 18572333

[114] Goodnow CC. Multistep pathogenesis of autoimmune disease. Cell. (2007) 130:25–35. 10.1016/j.cell.2007.06.033 17632054

[115] Goronzy JJ Weyand CM. Immune aging and autoimmunity. Cell Mol Life Sci. (2012) 69:1615–23. 10.1007/s00018-012-0970-0 22466672 PMC4277694

[116] Pisetsky DS. Pathogenesis of autoimmune disease. Nat Rev Nephrol. (2023) 19:509–24. 10.1038/s41581-023-00720-1 37165096 PMC10171171

[117] Bonasia CG Abdulahad WH Rutgers A Heeringa P Bos NA. B Cell activation and escape of tolerance checkpoints: recent insights from studying autoreactive B cells. Cells. (2021) 10:1190. 10.3390/cells10051190 34068035 PMC8152463

[118] Westerberg LS Klein C Snapper SB. Breakdown of T cell tolerance and autoimmunity in primary immunodeficiency—lessons learned from monogenic disorders in mice and men. Curr Opin Immunol. (2008) 20:646–54. 10.1016/j.coi.2008.10.004 18955138 PMC2605935

[119] Askenasy N Kaminitz A Yarkoni S. Mechanisms of T regulatory cell function. Autoimmun Rev. (2008) 7:370–5. 10.1016/j.autrev.2008.03.001 18486924

[120] Shevyrev D Tereshchenko V. Treg heterogeneity, function, and homeostasis. Front Immunol. (2020) 10:3100. 10.3389/fimmu.2019.03100 31993063 PMC6971100

[121] Macian F Im SH Garcia-Cozar FJ Rao A. T-cell anergy. Curr Opin Immunol. (2004) 16:209–16. 10.1016/j.coi.2004.01.013 15023415

[122] Zheng Y Zha Y Gajewski TF. Molecular regulation of T-cell anergy. EMBO Rep. (2008) 9:50–5. 10.1038/sj.embor.7401138 18174897 PMC2246614

[123] Liu Y Linsley PS. Costimulation of T-cell growth. Curr Opin Immunol. (1992) 4:265–70. 10.1016/0952-7915(92)90075-P 1418704

[124] Appleman LJ Boussiotis VA. T cell anergy and costimulation. Immunol Rev. (2003) 192:161–80. 10.1034/j.1600-065X.2003.00009.x 12670403

[125] Smith-Garvin JE Koretzky GA Jordan MS. T cell activation. Annu Rev Immunol. (2009) 27:591–619. 10.1146/annurev.immunol.021908.132706 19132916 PMC2740335

[126] Cabezas R Avila M Gonzalez J El-Bacha RS Baez E Garcia-Segura LM. Astrocytic modulation of blood brain barrier: perspectives on Parkinson's disease. Front Cell Neurosci. (2014) 8:211. 10.3389/fncel.2014.00211 25136294 PMC4120694

[127] Sonar SA Lal G. Blood-brain barrier and its function during inflammation and autoimmunity. J Leukoc Biol. (2018) 103:839–53. 10.1002/JLB.1RU1117-428R 29431873

[128] Windsor R Stewart SD Talboom J Lewis C Naymik M Piras IS. Leukocyte and cytokine variables in asymptomatic Pugs at genetic risk of necrotizing meningoencephalitis. J Vet Intern Med. (2021) 35:2846–52. 10.1111/jvim.16293 34687084 PMC8692191

[129] Toda Y Matsuki N Shibuya M Fujioka I Tamahara S Ono K. Glial fibrillary acidic protein (GFAP) and anti-GFAP autoantibody in canine necrotising meningoencephalitis. Vet Rec. (2007) 161:261–4. 10.1136/vr.161.8.261 17720962

[130] L.F. Eng. Glial fibrillary acidic protein (GFAP): the major protein of glial intermediate filaments in differentiated astrocytes. J Neuroimmunol. (1985) 8:203–14. 10.1016/S0165-5728(85)80063-1 2409105

[131] Tizard IR. Autoimmune Diseases in Domestic Animals. St. Louis, MI: Elsevier (2023). 10.1016/B978-0-323-84813-8.00032-5

[132] Middeldorp J Hol EM. GFAP in health and disease. Prog Neurobiol. (2011) 93:421–43. 10.1016/j.pneurobio.2011.01.005 21219963

[133] Maiolini A Carlson R Schwartz M Gandini G Tipold A. Determination of immunoglobulin A concentrations in the serum and cerebrospinal fluid of dogs: an estimation of its diagnostic value in canine steroid-responsive meningitis-arteritis. Vet J. (2012) 191:219–24. 10.1016/j.tvjl.2010.12.018 21277241

[134] Schwartz M Puff C Stein VM Baumgartner W Tipold A. Pathogenetic factors for excessive IgA production: Th2-dominated immune response in canine steroid-responsive meningitis-arteritis. Vet J. (2011) 187:260–6. 10.1016/j.tvjl.2009.12.001 20117950

[135] Maiolini A Carlson R Tipold A. Toll-like receptors 4 and 9 are responsible for the maintenance of the inflammatory reaction in canine steroid-responsive meningitis-arteritis, a large animal model for neutrophilic meningitis. J Neuroinflammation. (2012) 9:226. 10.1186/1742-2094-9-226 23016675 PMC3488568

[136] Fellman CL Archer TM Stokes JV Wills RW Lunsford KV Mackin AJ. Effects of oral cyclosporine on canine T-cell expression of IL-2 and IFN-gamma across a 12-h dosing interval. J Vet Pharmacol Ther. (2016) 39:237–44. 10.1111/jvp.12280 26676223 PMC4834224

[137] Leonard WJ Wan CK. IL-21 Signaling in Immunity. F1000Res. (2016) 5:7634. 10.12688/f1000research.7634.1 26966515 PMC4770986

[138] Suzuki M Uchida K Morozumi M Hasegawa T Yanai T Nakayama H. A comparative pathological study on canine necrotizing meningoencephalitis and granulomatous meningoencephalomyelitis. J Vet Med Sci. (2003) 65:1233–9. 10.1292/jvms.65.1233 14665754

[139] Matsuki A Takahashi M Yaegashi A Tamahara S Ono K. Serial Examinations of Anti-GFAP autoantibodies in cerebrospinal fluids in canine necrotizing meningoencephalitis. J Vet Med Sci. (2009) 71:99–100. 10.1292/jvms.71.99 19194083

[140] Shibuya M Matsuki N Fujiwara K Imajoh-Ohmi S Fukada H Pham NT. Autoantibodies against glial fibrillary acidic protein (GFAP) in cerebrospinal fluids from pug dogs with necrotizing meningoencephalitis. J Vet Med Sci. (2007) 69:241–5. 10.1292/jvms.69.241 17409638

[141] Matsuki N Fujiwara K Tamahara S Uchida K Matsunaga S Nakayama H. Prevalence of autoantibody in cerebrospinal fluids from dogs with various CNS diseases. J Vet Med Sci. (2004) 66:295–7. 10.1292/jvms.66.295 15107560

[142] S. Romagnani. Type 1 T helper and type 2 T helper cells: functions, regulation and role in protection and disease. Int J Clin Lab Res. (1992) 21:152–8. 10.1007/BF02591635 1687725

[143] Noelle RJ Snow EC. T helper cell-dependent B cell activation. The FASEB journal. (1991) 5:2770–6. 10.1096/fasebj.5.13.1833257 1833257

[144] Lemke L Carlson R Warzecha K Volk A Tipold A Nessler J. Elevated Interleukin-31 levels in serum of dogs with steroid-responsive meningitis-arteritis suggests an involvement in its pathogenesis. Animals (Basel). (2023) 13:2676. 10.3390/ani13162676 37627467 PMC10451616

[145] Guerrero-Garcia Jd Rojas-Mayorquin AE Valle Y Padilla-Gutierrez JR Castaneda-Moreno VA Mireles-Ramirez MA. Decreased serum levels of sCD40L and IL-31 correlate in treated patients with Relapsing-Remitting Multiple Sclerosis. Immunobiology. (2018) 223:135–41. 10.1016/j.imbio.2017.10.001 29050818

[146] Fleck TJ Norris LR Mahabir S Walters RR Martinon O Dunham SA. Onset and duration of action of lokivetmab in a canine model of IL-31 induced pruritus. Vet Dermatol. (2021) 32:681–e182. 10.1111/vde.12943 33830571 PMC9291138

[147] Krautmann M Walters RR King VL Esch K Mahabir SP Gonzales A. Laboratory safety evaluation of lokivetmab, a canine anti-interleukin-31 monoclonal antibody, in dogs. Vet Immunol Immunopathol. (2023) 258:110574. 10.1016/j.vetimm.2023.110574 36842258

[148] Ballabh P Braun A Nedergaard M. The blood-brain barrier: an overview: structure, regulation, and clinical implications. Neurobiol Dis. (2004) 16:1–13. 10.1016/j.nbd.2003.12.016 15207256

[149] Claudio L Raine CS Brosnan CF. Evidence of persistent blood-brain barrier abnormalities in chronic-progressive multiple sclerosis. Acta Neuropathol. (1995) 90:228–38. 10.1007/BF00296505 8525795

[150] Jackson RJ Meltzer JC Nguyen H Commins C Bennett RE Hudry E. APOE4 derived from astrocytes leads to blood–brain barrier impairment. Brain. (2022) 145:3582–93. 10.1093/brain/awab478 34957486 PMC9586546

[151] Wolburg-Buchholz K Mack AF Steiner E Pfeiffer F Engelhardt B Wolburg H. Loss of astrocyte polarity marks blood–brain barrier impairment during experimental autoimmune encephalomyelitis. Acta Neuropathol. (2009) 118:219–33. 10.1007/s00401-009-0558-4 19533155

[152] Alvarez JI Cayrol R Prat A. Disruption of central nervous system barriers in multiple sclerosis. Biochim Biophys Acta. (2011) 1812:252–64. 10.1016/j.bbadis.2010.06.017 20619340

[153] Bennett J Basivireddy J Kollar A Biron KE Reickmann P Jefferies WA. Blood-brain barrier disruption and enhanced vascular permeability in the multiple sclerosis model EAE. J Neuroimmunol. (2010) 229:180–91. 10.1016/j.jneuroim.2010.08.011 20832870

[154] Day MJ Histopathology Histopathology of EAE. In: Lavi E, Constantinescu CS. Editors. Experimental Models of Multiple Sclerosis. Boston, MA, USA: Springer (2005). p. 25–43. 10.1007/0-387-25518-4_3

[155] Lassmann H Suchanek G Ozawa K. Histopathology and the blood-cerebrospinal fluid barrier in multiple sclerosis. Ann Neurol. (1994) 36:S42–6. 10.1002/ana.410360713 8017888

[156] Hanael E Baruch S Chai O Nir Z Rapoport K Ruggeri M. Detection of blood-brain barrier dysfunction using advanced imaging methods to predict seizures in dogs with meningoencephalitis of unknown origin. J Vet Intern Med. (2022) 36:702–12. 10.1111/jvim.16396 35285550 PMC8965229

[157] Schwartz M Puff C Stein VM Baumgartner W Tipold A. Marked MMP-2 transcriptional up-regulation in mononuclear leukocytes invading the subarachnoidal space in aseptic suppurative steroid-responsive meningitis-arteritis in dogs. Vet Immunol Immunopathol. (2010) 133:198–206. 10.1016/j.vetimm.2009.08.007 19733404

[158] Püschel ML Freise F Carlson R Tipold A Nessler J. The Reibergram for immunoglobulin A in dogs: evaluation of intrathecal IgA synthesis using a quotient graph in dogs with neurological diseases. J Vet Intern Med. (2023) 37:191–203. 10.1111/jvim.16601 36507577 PMC9889711

[159] Rothschild MA Oratz M Schreiber SS. Serum albumin. Hepatology. (1988) 8:385–401. 10.1002/hep.1840080234 3281888

[160] Sorjonen DC. Total protein, albumin quota, and electrophoretic patterns in cerebrospinal fluid of dogs with central nervous system disorders. Am J Vet Res. (1987) 48:301–5. 3826872

[161] Weissberg I Wood L Kamintsky L Vazquez O Milikovsky DZ Alexander A. Albumin induces excitatory synaptogenesis through astrocytic TGF-beta/ALK5 signaling in a model of acquired epilepsy following blood-brain barrier dysfunction. Neurobiol Dis. (2015) 78:115–25. 10.1016/j.nbd.2015.02.029 25836421 PMC4426044

[162] Nadal A Fuentes E Pastor J McNaughton PA. Plasma albumin is a potent trigger of calcium signals and DNA synthesis in astrocytes. Proc Natl Acad Sci. (1995) 92:1426–30. 10.1073/pnas.92.5.1426 7877995 PMC42532

[163] Ralay Ranaivo H Wainwright MS. Albumin activates astrocytes and microglia through mitogen-activated protein kinase pathways. Brain Res. (2010) 1313:222–31. 10.1016/j.brainres.2009.11.063 19961838 PMC2812578

[164] Friedman A Kaufer D Heinemann U. Blood–brain barrier breakdown-inducing astrocytic transformation: novel targets for the prevention of epilepsy. Epilepsy Res. (2009) 85:142–9. 10.1016/j.eplepsyres.2009.03.005 19362806 PMC3615244

[165] Hassel B Iversen EG Fonnum F. Neurotoxicity of albumin in vivo. Neurosci Lett. (1994) 167:29–32. 10.1016/0304-3940(94)91020-0 7909931

[166] Blauth K Zhang X Chopra M Rogan S Markovic-Plese S. The role of fractalkine (CX3CL1) in regulation of CD4(+) cell migration to the central nervous system in patients with relapsing-remitting multiple sclerosis. Clin Immunol. (2015) 157:121–32. 10.1016/j.clim.2015.01.001 25596452

[167] Spitzbarth I Schenk HC Tipold A Beineke A. Immunohistochemical characterization of inflammatory and glial responses in a case of necrotizing leucoencephalitis in a French bulldog. J Comp Pathol. (2010) 142:235–41. 10.1016/j.jcpa.2009.08.158 19815229

[168] Miyake H Inoue A Tanaka M Matsuki N. Serum glial fibrillary acidic protein as a specific marker for necrotizing meningoencephalitis in Pug dogs. J Vet Med Sci. (2013) 75:1543–5. 10.1292/jvms.13-0252 23856761 PMC3942992

[169] Hanael E Chai O Konstanitin L Gibeon L Rapaport K Ruggeri M. Telmisartan as an add-on treatment for dogs with refractory idiopathic epilepsy: a nonrandomized, uncontrolled, open-label clinical trial. J Am Vet Med Assoc. (2022) 260:735–40. 10.2460/javma.20.12.0683 35201995

[170] Gonçalves R Decker SD Walmsley G Maddox TW. Prognosis in meningoencephalitis of unknown origin in dogs: Risk factors associated with survival, clinical relapse, and long-term disability. J Vet Intern Med. (2024) 38:1583–90. 10.1111/jvim.17037 38483069 PMC11099754

[171] Vineis P. Definition and classification of cancer: monothetic or polythetic? Theor Med. (1993) 14:249–56. 10.1007/BF00995166 8259531

[172] Xavier J Bursztejn C Stiskin M Canitano R Cohen D. Autism spectrum disorders: An historical synthesis and a multidimensional assessment toward a tailored therapeutic program. Res Autism Spectr Disord. (2015) 18:21–33. 10.1016/j.rasd.2015.06.011

